# Mothers explanatory models of infant stress & adversity in rural Haryana, India: qualitative findings from the Early Life Stress sub-study of the SPRING cluster-randomised controlled trial (SPRING-ELS)

**DOI:** 10.12688/wellcomeopenres.14943.1

**Published:** 2018-12-03

**Authors:** Sunil Bhopal, Deepali Verma, Reetabrata Roy, Gauri Divan, Zelee Hill, Betty Kirkwood

**Affiliations:** 1 Faculty of Epidemiology & Population Health, London School of Hygiene & Tropical Medicine, London, UK; 2 Northern School of Paediatrics, Newcastle upon Tyne, UK; 3 Sangath, New Delhi, India; 4 Institute for Global Health, University College London, London, UK

**Keywords:** toxic stress, adversity, child development, explanatory model, qualitative, health inequalities

## Abstract

**Background   **Exposure to a range of biological and psychosocial adversities in early childhood is of negative consequence through the lifecourse. This is particularly important for children in low- and middle-income countries where at least 250 million children are at high-risk of not meeting their developmental potential. Minimal evidence describes mothers’ views of this. We therefore elicited an explanatory model exploring mothers’ perceptions of infant stress and adversity in rural Haryana, India.

**Methods **We did eight focus-group discussions to explore the perspectives of mothers in the general population of this rural area of India using a discussion guide based on Kleinman’s explanatory model. Data were coded by two analysts and arranged in themes for presentation. Illustrative quotations were used for presentation of findings.

**Results **All mothers identified several causes of adversity and stress for children, including poverty, neglect and violence. They described the consequences of this for emotions, behaviour and school readiness of children, and that some of the consequences were reversible with appropriate management. Mothers described younger children as being unable to be affected by adversity, because they were “too young to understand”.

**Conclusions **Mothers agreed with much of the current biomedical model for early childhood development, however the predominant view was that young infants were “too young to understand” is an important deviation. These findings are of importance in designing behaviour change strategies for this crucial period of early childhood which is rising up the global policy agenda with the aim of giving every child the opportunity to thrive.

## Background

Being exposed to a wide-range of biological and psychosocial adversities including violence, neglect, and maternal depression in early childhood is of negative consequence across the lifecourse ^1^. In the absence of high-quality protective caregiving, exposure to these adversities is associated with a condition of toxic stress leading to biological changes throughout the body including overactivation of the sympathethic nervous system, excess of stress hormones, and systemic inflammation ^2^. This toxic stress is associated with both structural and functional changes in the brain, with implications for development, health, and disease throughout child- and adulthood ^3^. This is a concern worldwide, but particularly for those children growing up in the most disadvantaged settings; at least 250 million children in low- and middle-income countries are at high-risk of not meeting their developmental potential because of early childhood adversity ^4^. The president of the World Bank recently commented on the problems this poses for individuals and society:

*“There can be no equality of opportunity without…appropriate stimulation, nurturing, and nutrition for infants and young children. Conditions of poverty, toxic stress and conflict will have produced such damage that [children] may never be able to [take up] future opportunities. If your brain won't let you learn and adapt in a fast changing world, you won't prosper and, neither will society.” ^5^*

There is mounting momentum to improve this situation. The World Health Organization recently published a roadmap to improvement in its ‘Nurturing Care Framework’ for early childhood development ^6^. The ‘Nurturing care’ concept is made up of five interrelated components: adequate nutrition, good health, opportunities for early learning, security and safety, and responsive caregiving ^7^. Promoting this at an individual child level requires a meaningful engagement with families, particularly with mothers who are responsible for much of young-child caregiving. This study is based on the premise that mothers have their own explanations for the ways in which infants are growing and developing, and for the positive and negative influences of the environment, including adversity. These explanations influence a mother’s behaviour and therefore their capacity to consider new ideas from interventions aiming to improve early childhood development. There is minimal evidence describing ways in which mothers consider adversity and its association with child health and development and so the aim of this study was to elicit an explanatory model, exploring mothers’ perceptions of infant stress and adversity in rural Haryana, India. 

## Methods

### Setting

This study was conducted in Rewari district, Haryana state, India in September and October 2015. The district is predominantly rural, and has health and demographic indicators around average for the state. The literacy rate in Haryana at the last census was 76%, with female literacy of 67% ^8^. Infant mortality was 36/1000 live births – around the national average ^9^. More than one third of under-five year old children were stunted (extremely low height-for-age) ^10^. The district capital is Rewari town, which houses the district government administration, government program offices, and key healthcare facilities including the district hospital. Rewari town is around 90km from the Indian capital New Delhi, to which it is connected by rail and road highway. There are considerable family, cultural, and social connections between the villages of Rewari district, the town and surrounding area including New Delhi. Most villages have members who work and travel between the village and the surrounding urban areas. The study setting is diverse and families live in a variety of configurations, however the traditional ‘joint-family’ where multiple siblings (usually brothers) live with their wives, children, and parents is common. Children are therefore often raised by several adult caregivers, including parents, aunts/uncles, and grandparents.

The site is part of the SPRING (Sustainable Program Incorporating Nutrition and Games) programme, a home visits intervention delivered at scale and aiming to improve early child growth and development. SPRING is evaluated by cluster randomised controlled trial and this is described in detail elsewhere (clinicaltrials.gov registration NCT02059863; see SPRING website).

### Choice of data collection method and sampling

We selected focus-group discussions as the most appropriate method by which to explore the combined perspectives of mothers. Collecting data in this way in groups allowed for interaction between participants, and for new thoughts to emerge from discussion. This was important because we were not exploring a named disease but a concept which was not clearly familiar to all participants at the start of discussions. The groups allowed us to gain a broad understanding of models of child development by which we could gather explanations, reasons behind these, and tensions between them. We also wanted to develop insight into which elements were widely shared, and which were more open to individual interpretation.

We used purposive sampling to identify mothers of any age and background, with at least one child aged under 2 years, who lived in the control clusters of the SPRING study area across three blocks of Rewari District, Haryana, India (i.e they were not receiving SPRING home visits). Participants were excluded if they were unable to speak Hindi, or if they were not able to travel to the focus group location. They were included based on their ability to contribute their knowledge on early childhood, and fieldworkers selected participants who they felt were willing and able to share their experiences and views in a group setting. We ensured that they had lived in the area for the majority of their young child’s life. This is because some mothers move between their parental- and family- homes when children are young, and we wanted to understand the perspectives of those most settled in the study area as this comprises the majority of the population. A SPRING resident fieldworker with knowledge of the local area and families identified participants who met these criteria and were willing to take part. The mothers selected were from a range of ages and were broadly representative of the communities in which they live. Fieldworkers did not report difficulties recruiting mothers and none dropped out after agreeing to participate.

### Focus group methods

We prepared a discussion guide based on Kleinman’s explanatory model ^11^, conceptualising illness within a framework comprising aetiology, onset, consequences, prognosis, and treatment. Kleinman notes the difference in illness explanation between lay-people and professionals, and builds on Engel’s position that the biomedical model focusses on anatomy and biochemistry over information, beliefs and concepts ^12^. In this study we specifically wanted to identify areas of conflict between mothers and biomedical explanations.

We wrote the guide in English, translated it to Hindi and then did a back-translation to assess accuracy (see Extended data ^13^). We pre-tested the guide with six SPRING staff members who are local residents.

The guide started with two warm-up exercises. These aimed to: introduce the general area of discussion, establish participants as experts compared to the moderator, and introduce planned methods of probing. Mothers were asked to group 20 pictures of child facial expressions into 4 piles: “happy”, “sad”, “neutral” and “cannot tell” - they were then asked to comment on their own and peers decisions, and were challenged to explain and expand on this thought process. In the second exercise mothers were asked to sort a pile of pictures of objects into piles of items that make a child happy, sad or has no effect (images available as Extended data ^13^).

The next step was outlining two scenarios. The first was of a family living in poverty with low-income, poor-quality housing, and overcrowding. Mothers were asked to comment on possible consequences for a child living in this family. Following discussion, the second scenario was introduced illustrating a family with a mother with alcoholic-use problems and a mother with depression. The guide then outlines topics relating to ways in which these scenarios affect children, timing of this effect, other potential causes, and other longer-term consequences for children. Finally, the guide outlines a discussion around prevention and treatment. The moderator was encourage to modify questions and question-order as appropriate to the sessions.

Sessions were conducted at Anganwadi centres or in a health sub-centre (lower level health system facilities), in order that participants were able to easily access the venue and were attending a socially acceptable meeting point. Participants usually lived in the village in which the session was held, and occasionally in a nearby village. In the vast majority of cases, they did not know each other prior to the session, and had not met the moderator previously. We estimated that 5-10 sessions would be required in order to reach a theoretical point of data ‘saturation’ based on our prior experiences. We expected this to depend on the degree to which the discussion guide and moderation made the topic accessible to participants.

Following piloting with a group of mothers who worked in the SPRING programme, seven focus groups were held, each with 4-6 participants. We felt that given the context and topic, these relatively small groups would encourage active contributions, and that they would facilitate high quality interactions between participants. Each group lasted 50-65 minutes. A total of 34 mothers aged 20 to 35 years took part. Data were collected between September and October 2015 by a female, local research associate (DV) with a PhD in physical anthropology, who was familiar with the local culture and language. DV was trained in qualitative methods, and was well acquainted with the subject, study objectives and discussion guide. DV moderated the focus groups using the guide for structure, and was encouraged to modify questions and order of these as appropriate to moderate high-quality discussion.

### Data analysis

Field notes were taken by the moderator who later listened to audio recordings and expanded these, recording her own understanding of the session through reflexive writing and also recording participant’s direct quotations in Hindi alongside a translation into English. These ‘expanded notes’ give a strong reflection of the details of a session in the context of the moderator’s comments, and observations of participants and group dynamics ^14^. Each draft was discussed in detail with SB and finalised whilst listening to the recording of the session. These expanded notes were the data with which analysis was performed.

We aimed to discover and build an explanatory model by using several components of the grounded theory. There was no *a priori* hypothesis. Data were collected, analysed, provisionally coded, and understood during the process of data collection. Each element of analysis altered subsequent FGDs and the moderator included current understanding in probes and discussions to tested emerging theories with new participants.

Data were continuously analysed by reading the expanded notes, and creating initial word-processed tables capturing data from the expanded notes within provisionally labelled themes. After three sessions we carried out a fuller analysis to identify gaps in understanding, to check if saturation had been reached, and to evaluate the themes and sub-themes that were emerging. After three further sessions (session six) data saturation was approaching as few new concepts were emerging. The data was rich, varied and grounded in what the participants had discussed. In the seventh session, the moderator found that she was able to use fewer prompts, and that the data was in accordance with the synthesised findings of sessions 1-6.

Following the final session, DV and SB read through all data several times on one laptop computer for familiarisation and used NVIVO 11 (QSR international), to code data into domains. Data could be coded in zero, one or more than one domain. Next a document containing all data separated into domains was printed. Each line was analysed in an attempt to ‘fracture’ the data to open up multiple lines of enquiry, compare with existing theories, and understand the deeper meaning of the data, and coded zero, one or more codes. Many codes were generated at this stage - the aim was to consider the data as fully as possible, and many of these were later amalgamated or discarded.

Following this fracturing of the data, the codes were arranged within themes to generate themes and subthemes, which is the way in which the data are presented in this report. At this stage there were many quotations per sub-theme. The most descriptive quotations were chosen to represent sub-themes and are presented to illustrate the findings of data analysis.

### Ethics

The Institutional Review Board of Sangath (27 May 2015) and the Research Ethics Committee at The London School of Hygiene & Tropical Medicine (19 May 2015, approval number 9886) reviewed and approved the study. Participants were approached in their home by the SPRING fieldworker and DV in the days preceding a planned session. At the beginning of each focus group, an information sheet was read out by DV and participants were invited to discuss this. Participants were asked to keep the session confidential and were assured that the recordings and fieldnotes would be stored securely and not shared with anyone. Written informed consent was then obtained from all participants for both participation and audio-recording of the session. Participants were informed that the study was being conducted for the purposes of understanding how people in the geography think about infant wellbeing. All data were kept on password protected disk drives, data were anonymised for analysis and audio recordings will be deleted one year after publication.

## Results

All mothers in all groups recognised both initial scenarios, and were able to describe similar individuals and situations they had come across previously. All participants agreed that the outlined scenarios could cause harm of some type to children. All participants contributed actively, being keen to contribute their thoughts on this topic which related strongly to their own experiences of raising young children. The word ‘ *tanau’* was used to describe ‘stress’ in Hindi. This term captures a condition which participants described both adults and children as having the potential to suffer from. There was no regularly used word to describe ‘adversity’.

Participants explanatory models of childhood stress and adversity are presented in Table 1, organised into four major themes: causes, mechanisms and consequences, prevention and treatment. Three of these have sub-themes.

**Table 1.** Explanatory model for childhood stress and adversity - themes and subthemes.

Theme	Sub-theme
Causes	Child, Family and Community Causes
Mechanisms and Consequences	Age of “understanding” Mechanisms connecting adversity to consequences Early emotional and behavioural consequences Longer term consequences Reversibility
Prevention	Avoiding stressors Adult support
Treatment	Supportive adult care Medical professionals

### Causes

Each mother identified several causes of adversity and stress. Most were discussed in several FGDs and are listed with illustrative quotations in Table 2. The table is listed in order of frequency in which the cause was discussed.

**Table 2.** Causes of childhood adversity and stress identified by participants in focus group discussions.

Cause	Illustrative quotes
Poverty	*“when a child gets hungry....or only gets food half of the time....that child wishes for everything they see,* *thinking that if they can get it, they can eat” FGD 5* *“In a poor family proper attention is not given to a child, there is no food, there is a lack of money, there is* *angriness…a child is often beaten” FGD 6*
Neglect	*“If proper attention is not given to young children, such as inadequate feeding, playing or talking with the* *mother, then the child will not be healthy....they’ll lie down silently, and not pay attention to things happening* *around them” FGD 4*
Violence	*“[when there is alcoholism and fighting in the household] the amount of attention a mother can pay towards* *looking after her child is reduced” FGD 2*
Pregnancy maternal mental health including stress	*“When a mother thinks about negative things whilst pregnant, it has a negative impact on the unborn child....* *on their mental wellbeing....they might be born weak” FGD 2* *“In local language it is said that when child is in the womb, mother should not take stress....as we say that it* *affects the child in the womb” FGD 5* *“when the mother is fine she will be able to take care of child, when she is sad how will she take care?” FGD 6*
Alcoholic father	Commonly discussed in responses to initial scenarios, with universal agreement that this can cause problems for children.
Household environment	*“Being unable to play because of living in a crowded space will have physical and mental effects” FGD 4*
Birth order	*“Sometimes what happens is a mother has a workload of two children.... When it was only one she [was able* *to] give complete care....but now when she has to give attention to both children, she takes time to adjust....* *” FGD2*

Mothers were clear that the effect of each adversity varies from child to child.

*“We never know which child will take what to heart....maybe there has been an incident which has occupied their mind.....resulting in [unusual] behaviour. We often do not know what might have hurt a child. If it had been considered during childhood, maybe behaviour would have been different” FGD 1*

Contrary to this negative view, occasionally in several groups individual mothers argued that adversity can lead to positive consequences.

*“A poor child would be very well behaved, as he has more understanding about valuing the things and opportunities he has, compared with a child born into a rich family” FGD 3*

### Mechanisms and consequences

***Age of “understanding”.*** The age at which children are seen to be able to “understand” is the crucial factor rather than a specific age in years. Children were described as being shielded from the impact of adversity and stress until they are able to process and comprehend what is happening in their environment. When asked directly by the moderator to give an age, most participants agreed that this was around 3 or 4 years of age.

*“if he is too young he will not have stress, only if he understands things then he can become stressed....a 4 year old child is able to understand” FGD 7*
*“young children do not know what is happening… only when they understand do they know what is happening [regarding violence or maternal distress] and then it can affect them” FGD 6*
*“two year old children do not understand anything, they are happy and play…there won’t be any effect [of adversity] at this age - at 4–5 years old, that’s when they start to understand” FGD 3*

During one discussion, a mother pointed to her own baby situated in her lap and said:

*“he does not know anything – these things [adversity] don’t make him cry, don’t make him sleep…it makes no difference” FGD 2*

Even if household difficulties are noticed, children were described as being quick to forget, giving another reason why they cannot be impacted by adversity.

*“a younger child will forget as quickly as he learns…” FGD 4*
*“Younger children do not get bothered, a 3–4 year old child is able to understand the things and for a moment things will be in their mind....later when they play, they will forget about them and move on. Even if a younger child is beaten, after sometime he again gets involved in games” FGD 1*

Contrary to this, a few mothers described young children being affected whilst young – but this was not a predominant view.

*“[mothers] need to give proper care until 2 years of age… because at this stage physical and mental development occurs” FGD 4*
*“…young children learn quickly and remember....whereas elder children understand [and are able to think], so may or may not be affected by adversity” FGD 3*

***Mechanisms connecting adversity to consequences.*** Two key mechanisms were discussed. First, adversity was described as leading to changes in the household environment and caregiver capacity, both of which are related to negative consequences for children. Second, the brain was described as a key way in which adversity more directly translates into physical and mental health problems over the lifecourse. This was understood in several ways but most commonly, ruminating on hardship (or adversity ‘staying in the mind’) was described as being bad for the brain.

*“[The impact of adversity] is felt directly on the brain” (all participants in unison)… “[adversity] then remains in the child’s mind” FGD 2*
*“for instance if there is a fight the child trembles, starts crying and there is major effect on the child’s heart....these negative instances remain in a child’s mind and go on to affect their brain…[this is] because these instances will keep wandering in their mind, even when the child is playing” FGD 5*

The transmission of adversity was additionally described as beginning from conception, with significant importance placed on the *in-utero* period. Mothers described ‘blood-mixing’ between mother and child and the umbilical cord and its role in connecting the mother and foetus.

*“it is said that [mother and child connect] through the umbilical cords. When the cords touch each other, mother and child blood mixes” FGD 2*
*“If a mother is tense then that will have a direct impact on her child’s brain during pregnancy” FGD 3*

There was a suggestion in several discussions that children who have constant adversity may appear to be coping better than those with unpredictable environments.

*"when fighting in the household is rare, children cry and are unhappy. But when these things become a daily habit, children get used to them. If the child continues to pay attention to the negativity, they might be sad – maybe crying, staying quiet, or not playing, but usually they do normal things” FGD 5*

***Early emotional and behavioural consequences.***

***Physical and mental development***

Adversity was widely described as leading to *Kamjori.* This phrase describes a physical and mental ‘weakness’ including growth stunting, lethargy, intellectual impairment, cognitive deficiencies and development delays.

*“[children living with adversity have] hampered mental and physical growth....the brain will not grow and neither will the body. When the child plays with other children he will be kamjor and get tired easily” FGD 5*

***Low activity level***

These children were described as being less active than others and being less willing to play. This was described as causing further physical and mental problems.

***Emotions and behaviours***

In the short term, adversity was linked to moodiness, crying, sadness, anger, violence and jealousy.

*“[The child] stays sad, even when playing with other children in neighbourhood and develops low self esteem” FGD 3*
*“[a child in adverse circumstances] will get angry....sad....be worried for the whole day....they will get upset easily”. FGD 3*

Discussion showed adversity being connected with children being nervous and fearful. This was linked most often to those children living in homes with violence or where the father was seen as being unpredictable (often linked to alcohol consumption).

*“[my child] gets frightened.... he fears that he would be scolded as his mother is being scolded and this makes him unhappy…” FGD 2*

Mothers were clear, however, that these behaviours are not only caused by adversity and that it is not possible for a mother to know exactly what has caused their child’s problem.

***Longer term consequences.*** Mothers recognised that earlier consequences may continue into later childhood and adulthood causing further consequences for physical and mental health.

*“earlier effects will remain - if the child’s situation does not improve with time then these will continue” FGD 4*

Poor school readiness and inferior academic performance during education is a long term consequence of early life adversity.

*“When the child grows, he will face problem in his studies as in the beginning of school he can’t learn ABCD properly - without this how can he proceed further?” FGD 3*

Participants described a cycle of adversity – for example, those children brought up by an alcoholic father are more likely to misuse alcohol themselves in later.

*“When (the child) sees father drinking, he will learn the same habit and this will cause him [problems as an adult]” FGD4*

***Reversibility.*** Mothers described improvements in adverse circumstances leading to reduced consequences for children, with the caveat that there may be an enduring impact, particularly for some of the more severe adversities -

*“when a child receives love, it may reduce the negativity by some percentage but something will be left in the child’s mind that these things previously happened in the house, and something will remain” FGD 4*
*“if there is a proper improvement [in circumstances], the child may change but otherwise, childhood weakness can’t be recovered rapidly” FGD 5*

and that the earlier a stressor is removed the better because managing consequences becomes more more difficult over time.

*“Initially it’s a small thing, but later it could grow bigger....if it is found and dealt with when the child is small it is fine otherwise it would keep on amplifying and become a big problem” FGD 3*

### Prevention

***Avoiding stressors.*** Mothers detailed ways in which they try to protect their own children from stress and adversity. This mainly involved keeping them away from sources of stress.

*“Mothers avoid fighting in front of their children in order to stop household conflict affecting children” FGD 1*
*“Suppose a child’s father drinks [alcohol] - he should be kept away and the mother should play with the child. Even if there is a fight then after some moment, the mother should talk happily so that the child forgets what has just happened” FGD 5*

Mothers also described their own role in avoiding stress whilst pregnant.

*“Pregnant women should have food on time, walk around, avoid tension....the child in the womb should not suffer so that they can remain healthy” FGD 1*

***Adult support.*** The importance of care for supporting children in adversity was emphasised. This includes spending time and playing with children, and clearly showing them love and affection.

*“Mother should take out time from household work to give attention to the child, she should spare time to feed the child, this would enable a child to understand that their mother is there and to help them to feel safe” FGD 4*
*“Take out time for child, try to understand what the child has to say and is feeling....” FGD 3*

The question of who is most suitable for offering this care was repeatedly raised with mothers promoting the joint-family system and its benefits for caring. Family members including the grandmother and neighbours often extend their help.

*“Until 6 months of age a child is breastfed and so spends most of the time with his/her mother. As they grow older, other family members can take care – grandmother, grandfather....anyone in family can do this” FGD 1*

### Treatment

***Supportive adult care.*** Children suffering from the negative consequences of adversity are seen as being predominantly a family problem that should be treated by offering extra love and care.

*“Talk to the child with love, call them near, give them things....so that the child’s mind is distracted....” FGD 1*
*“The mother has to take care, by giving love....then they would be happy” FGD 2*
*“Give the child what they need - if they need food, give food – if they need to have a bath, give bathe ....” FGD 2*

However, mothers were clear about the difficulties faced in this regard if the adversity is continuing. Impacts last until the adversity stops or the family support improves.

*“Unless their parents stop fighting [the adverse consequences] will remain until adulthood” FGD4*
*“Only if circumstances become favourable, then a child’s tension can be reduced so that they have the capability to go ahead in life” FGD3*

***Medical professionals.*** Doctors were mentioned by several mothers as having a role, but they did not know what sorts of treatments or cures might be offered. One participant described a doctor asking for a test, but that in her experience this comes back normal for these sorts of children.

## Discussion

This study describes a qualitative exploration of mothers views regarding childhood adversity in rural Haryana, India. It is, to the best of our knowledge, the first such study in a low/middle-income country setting.

Mothers’ explanations of the effects of adversity and stress were broadly in alignment with biomedical understanding on the wide range of adversities faced by children, their potential consequences and adult care as the key to prevention and treatment. This is in accordance with the literature on adverse childhood experiences from high-income settings, where increased number of adversities are associated with impaired development and emotional regulation ^15, 16^, later-life depression ^17^, and causes of death in adulthood including cardiovascular disease, cancer and diabetes to name a few ^18^. It is also in accordance with the focus on promotion of high-quality parent-child interaction, maternal health and the mother-child relationship, through early childhood development policy and programmes. Mothers described adversity reflecting in emotional and behavioural consequences for children in the relatively short term and there was broad agreement that these consequences can persist into later child- and adulthood with reversal becoming increasingly difficult.

The brain was described as being a link between adversity and consequences. This is in accordance with currently biomedical understanding on the impacts of ‘toxic stress’ on the developing brain, and messaging being used in some high income countries to build communities of practice aiming to improve childhood development (for example, in the USA, the National Scientific Council on the Developing Child ^19^).

There were important exceptions to this concordance. Of particular note, the widely held view view of young children being “too young to understand” is at odds with neuroscientific and epidemiological evidence suggesting that adversity in this early-life period has negative consequences. This evidence underpins much of the currently early childhood development agenda, and so those developing early childhood development programmes may wish to test and address this potential barrier to implementation in their own context. This finding is not well-described in the literature, but has also been noted by programmers including in an Early Childhood Development programme funded by the Department for International Development in Zambia (R Hughes, LSHTM, personal communication. May 2018)

Interestingly, mothers did not mention preference for boy-children, highly prevalent in this setting which has a sex-ratio at birth of 879 females per 1000 males ^8^, and which leads to discrimination in allocation of healthcare and other resources throughout childhood ^20, 21^.

Mothers often used the word ‘ *kamjori’* to describe ‘weakness’. It is an aspiration of all mothers to avoid this weakness with regards to their children. Previous descriptions in the literature focus on its relation to undernutrition ^22, 23^ and the term, which describes a broad range of negative physical and mental health features of young children, may be of use in framing future interventions in this geography and similar terms are likely to be used elsewhere.

The study provides useful data on a relatively unexplored area. Strengths of our methodology include our structured approach to focus group methods, including that emerging descriptions were tested in focus groups throughout data collection, giving a sense of the degree to which participants agreed with our analysis. Using two analysts meant that disagreements in coding and interpretation were noted and dealt with early in the analysis process. A limitation of our use of focus-groups was that mothers mainly discussed children abstractly, rather than discussing personal experiences. Future work could compare these findings with those conducted through in-depth interviews, and also collect data from other key carers and family members. We attempted to overcome other limitations through high-quality moderation, including the potential for conformity bias where participants state opinions that go along with previous participants, rather than their own views.

## Conclusion

Improving the capacity of parents and other caregivers to provide optimal nurturing care is seen as being one of the major solutions to supporting all children to reach their developmental potential. Designing interventions that work depends on thoroughly understanding the ways in which caregivers understand child development and the impact of environmental factors including adversity on young children. Results from this study suggest that in this area of rural India, mothers are in agreement with much of the current biomedical model of early childhood, and that they may therefore be receptive to behaviour change messages that make use of these concepts. There were, however, crucial areas of divergence including that mothers believed that young infants were ‘too young to understand’. This is of great importance for those designing interventions aiming to improve development in this crucial period of early childhood where learning potential is at its peak and the impact of suboptimal care can be lifelong.

## Declarations

### Ethics approval

London School of Hygiene and Tropical Medicine (9886) and Sangath (27 May 2015)

## Data availability

The raw data are not provided because the audio and focus group transcripts contain identifiable and sensitive information regarding individual participants (including names and locations). These participants were assured that discussions would be kept confidentiality and we do not believe that providing open access to the raw data would uphold this assurance.

Researchers who wish to access the data for re-analysis or for integrity purposes can do so by contacting the corresponding author and this will be done on agreement of the Sangath Institutional Review Board (H No 45 Bhatkar Waddo, Porvorim, Goa 403501 India; contactus@sangath.in; +91 7887872345), LSHTM Research Ethics Committee (Keppel Street, London WC1E 7HT; ethics@lshtm.ac.uk) and the researcher's research ethics committee/institutional review board or equivalent. The key considerations will be the aim to minimise potential harms and to protect the interests of participants. Sangath will remain the custodian of all data which will be provided under the following conditions. Firstly, researchers must be able to guarantee confidentiality of participants. Second, the authors of the article must be informed about any publication (paper, theses, dissertations, presentations, among others) in which the data will be used, prior to their dissemination. Finally, if the data is used in any other publication, it should be acknowledged that it is of secondary source, providing the appropriate citation.

### Extended data

The study guide and images used in the warm-up exercise are available from the LSHTM Data Compass

LSHTM Data Compass: Extended data. SPRING Early Life Stress Sub-study: Additional Resources https://doi.org/10.17037/DATA.00000947 ^13^

Data is available under a CC BY-NC-SA 3.0 licence
