# Supplementary material for: Mothers explanatory models of infant stress & adversity in rural Haryana, India: qualitative findings from the Early Life Stress sub-study of the SPRING cluster-randomised controlled trial (SPRING-ELS)
Source: Wellcome Open Res. 2018 Dec 3;3:153. [Version 1] doi: 10.12688/wellcomeopenres.14943.1 (PMC6611137; doi:10.12688/wellcomeopenres.14943.1)
Supplement: Peer review history

# Peer review: Reviewer response for version 1

**DOI:** 10.21956/wellcomeopenres.16297.r35690

**Published:** 2019-07-03

**Authors:** Alessandra N. Bazzano

**Affiliations:** 1 Global Community Health and Behavioral Sciences, Tulane University School of Public Health, New Orleans, LA, USA

The authors are to be congratulated on their excellent work in this important area. The writing is excellent and the manuscript is very interesting to read. It also parallels work in other areas of the world and with other populations. A few revisions would allow the work to be more clearly understood. General comments:

1. It is recommended that the authors utilize, and specifically cite, the COREQ guidelines for reporting on qualitative research. It is key to more clearly articulate their underlying approach to the qualitative research - is this a grounded theory study, is this ethnography, narrative qualitative research, etc...? This is a requirement for the reader to understand the way the methods have been carried out and why. For example, it is not useful to know that there were 2 independent coders if we do not know how that relates to validity because we haven't been told what type of qualitative approach was used. This should include references in the Methods section to guiding qualitative approaches - is the grounded theory constructivist (Charmaz) or along the lines of Strauss and Glaser or other? Please inform the reader.
2. Please provide a sentence or two (in the Introduction and at the Discussion) to buffer the intensive focus on mothers perspectives when these are shaped and informed by family and community/sociocultural norms - the authors present the data as though mothers are solely responsible for their interpretations of child development, yet they also acknowledge the strong "family, cultural, and social connections" and state that "Children are often raised by several adult caregivers". A sentence or two to indicate that the authors are aware that mothers' individual understandings are linked to that of other adults and the community, and often transmitted through these and over the life course, would be sufficient.
3. The conclusion that mothers have come to, that young infants are "too young to understand" is actually correct on the part of mothers, because the word "understand" relates to a cognitive function. Are the authors sufficiently certain that this also meant that the mothers were stating that young infants were *unaffected *by the adversity on the basis of the data? If so, it would be important to distinguish the difference between *understanding* and *being impacted by* adversity, please address this with a sentence or two.
4. It is not entirely correct to state that there is "minimal evidence describing ways in which mothers consider adversity and its association with child health" and there is a large body of literature (albeit from other settings) which has been omitted. Suggest revising this statement and also including in the Introduction and Discussion at least a couple of references to available literature in this area - even if from high income settings since the authors mention these at the end of the manuscript anyway.

Specific sections:

1. Abstract: Replace the words "Minimal evidence" with "Minimal literature" or "Minimal research". There is also a grammatical issue in the Conclusion sentence.
2. Methods:
  a) remove the section that states the discussion guide was based on Kleinman's model as this conflicts with the previous statement that "we were not exploring a named disease but a concept".
  b) explain more about the 20 pictures of child facial expressions (are these local children, are they culturally relevant facial expressions? Please confirm). 
  c) why are the two scenarios relating to "mother with alcoholic-use problems" and "mother with depression" when the informants mention father with alcohol problems - is this a typo and should it be father with alcohol use problem (also shouldn't this properly be termed "problematic alcohol use" or "alcohol use disorder")?
  d) when noting that the data were "collected by DV with a PhD in physical anthropology", please recognize that this may create a social desirability bias or power imbalance between the highly educated researcher and informants. 
  e) please provide citation for references to Grounded Theory (Strauss and Glaser?) and theoretical saturation (see Saunders *et al.*, 2018 ^1^), as well as "data coding into domains" (?).
3. Results:
  a) under the section "Causes" are the authors suggesting that overall, informants said that adversity can lead to negative consequences? And if so please state that as that is not clear from the description in the start of the paragraph. 
  b) please check that quotes support assertions, and consider removing those that don't strongly/clearly support, replacing with a sentence or two paraphrasing.
4. Discussion:
  a) section needs more references, whether drawn from quantitative literature or high income countries, rather than simply stating "to the best of our knowledge, the first such study". 
  b) move the section noting that the area has a preference for boy-children with disproportionate sex ratio to the Methods section on study setting. 
  c) add the following to the Limitations section, having only participants who spoke Hindi and who could travel to the location may have excluded other perspectives.

I confirm that I have read this submission and believe that I have an appropriate level of expertise to confirm that it is of an acceptable scientific standard, however I have significant reservations, as outlined above.

## References

[1] : Saturation in qualitative research: exploring its conceptualization and operationalization. *Qual Quant*.2018;52(4) : 10.1007/s11135-017-0574-8 1893-1907 10.1007/s11135-017-0574-8 29937585PMC5993836

# Peer review: Reviewer response for version 1

**DOI:** 10.21956/wellcomeopenres.16297.r35741

**Published:** 2019-06-10

**Authors:** Abdelhamid J. Afana

**Affiliations:** 1 McGill University, Montréal, QC, Canada

The manuscript “ *Mothers explanatory models of infant stress & adversity in rural Haryana, India: qualitative findings from the Early Life Stress sub-study of the SPRING cluster-randomised controlled trial (SPRING-ELS)”*: the content of the manuscript is relevant and likely to be of interest to readers and the journal. I found the manuscript to be clearly written and would like to offer a few suggestions.

- What is the operational definition of “toxic stress”?
- Methodology needs more description and clarifications. I would appreciate more elaboration on the following:
   
  - How were the findings generated by the “two warm-up exercises” utilized in the next step that outlines two scenarios? In addition, are inclusion and exclusion criteria the same as described? How is it analyzed?
  - “ ... The next step was outlining two scenarios. The first was of a family living in poverty … ” how are these families selected (inclusion and exclusion criteria)? There is no demographic data illustrating families’ background.

- Are moderators trained to run focus group discussions? What is their professional and cultural background?
- What is the total of mothers registered in the control cluster of the SPRING? More elaboration on the demographic background of participants is important such as education, number of children, economic status, etc. It would be good to describe that in a table.
- What is the main spoken language in Rewari district? Do authors think that limiting the language to Hindi is considered one of the limitations of the study? It may lead to over/under-representation of mothers.
- The authors stated “… We wrote the guide in English, translated it to Hindi ...”. I would appreciate more elaborations on the following:
   
  - What are the reasons for writing the guide in English then translating it into Hindi? Why not write the guide in Hindi language from the start?
  - Translation gives statistical validity of the guide, how did the authors obtain the cultural validity of the guide?

- I would appreciate it if the authors give samples of guide interview questions used in the focus groups.
- I would appreciate the authors giving definitions to the themes and sub-themes before describing them.
- The links between brain and adversity are not clear - I would appreciate the authors giving more description highlighting the links; the framework is not clear to readers.

I confirm that I have read this submission and believe that I have an appropriate level of expertise to confirm that it is of an acceptable scientific standard, however I have significant reservations, as outlined above.

# Peer review: Reviewer response for version 1

**DOI:** 10.21956/wellcomeopenres.16297.r34574

**Published:** 2019-01-24

**Authors:** Giridhara R. Babu

**Affiliations:** 1 Indian Institute of Public Health (IIPH) Bangalore, Public Health Foundation of India, Bangalore, Karnataka, India

1. It is better to compare the evidence regarding influencers of infant stress and adversities in the family environment from elsewhere (including in high-income countries ) with that of the observations in this study. 
2. It is important to explore if there is any correlation between the educational background of the women and perspectives expressed in the FGDs.
3. What is the rationale behind choosing only focus-group discussions, and why not In-depth interviews to explore the attributes in detail?
4. It is unclear if the FGDs explored other perspectives; Some of them are as follows:
  - Perspectives from a single or divorced parent, widow/ widower, upbringing of the children by father etc. would have been helpful to give more insight on this topic.  
  - There are no perspectives provided about the role of gender preferences. Especially, if the perception of what is stressful is the same for female and male children; whether they are cared for similarly or differentially.
  - Mothers described young children less likely to get affected by adversity, as they were “too young to understand.” This can reflect either as a limitation of their understanding or paternalistic attitude of mothers. 
5. The psychosocial environment assessment is incomplete without addressing buffers, mediators, and factors interacting and alleviating stress. The role of these factors (such as social support) should have been presented similar to the causes presented in the table.2 The authors should attempt and try to provide a conceptual framework accounting antecedent, mediators and modifying factors alleviating the effect of stress. The title suggests that explanatory models of infant stressors would be provided, while the results contain only a few factors (and not complete model or mechanisms) 
6. Discussion section needs more elaboration regarding all the themes presented in the results of the study.

I confirm that I have read this submission and believe that I have an appropriate level of expertise to confirm that it is of an acceptable scientific standard.
